# Transcriptomic Insights into Gas6-Induced Placental Dysfunction: Gene Targets for Preeclampsia Therapy

**DOI:** 10.3390/cells14040278

**Published:** 2025-02-13

**Authors:** Matthew Jackson, Trenton M. Gibson, Ethan Frank, Garrett Hill, Benjamin Davidson, Paul R. Reynolds, Benjamin T. Bikman, Brett E. Pickett, Juan A. Arroyo

**Affiliations:** 1 Department of Microbiology and Molecular Biology, Brigham Young University, Provo, UT 84602, USA; 2 Department of Cell Biology and Physiology, Brigham Young University, Provo, UT 84602, USA

**Keywords:** Gas6, preeclampsia, transcriptomics, placental dysfunction, therapeutic target

## Abstract

Preeclampsia (PE) is a complex pregnancy-specific disorder characterized by hypertension, proteinuria, and systemic inflammation, posing significant risks to maternal and fetal health. This study investigates the role of growth arrest-specific protein 6 (Gas6) in PE pathogenesis using a rat model. Gas6 administration induces hallmark PE features, including hypertension, proteinuria, and significant alterations in placental gene expression. Transcriptomic analysis revealed changes in pathways related to extracellular matrix remodeling, interleukin signaling, and oxidative stress, highlighting their contribution to PE pathology. Key findings include the upregulation of Fam111a, linked to oxidative stress and DNA replication, and the downregulation of Clca4, associated with ion transport and cellular homeostasis. Protein-level validation through immunofluorescence confirmed these alterations, reinforcing their mechanistic roles in placental dysfunction. Enrichment analysis further identified significant disruptions in extracellular matrix organization and intercellular signaling. These results underscore the pivotal role of Gas6 in exacerbating placental oxidative stress and systemic inflammation. Importantly, therapeutic inhibition of the Gas6/AXL axis using small-molecule inhibitors mitigated PE-like symptoms, highlighting its potential as a therapeutic target. This study provides novel insights into the molecular underpinnings of Gas6-mediated placental dysfunction and supports the development of targeted therapies to improve PE outcomes.

## 1. Introduction

Preeclampsia (PE) is a complex, pregnancy-specific disorder defined by the onset of hypertension and proteinuria after 20 weeks of gestation. It affects 2–8% of pregnancies globally and remains a major cause of maternal and fetal morbidity and mortality [1]. The multifaceted nature of PE involves impaired trophoblast invasion, endothelial dysfunction, and heightened inflammatory responses, contributing to significant pregnancy complications. The disease originates from abnormal placental development, where trophoblasts fail to invade maternal spiral arteries adequately. This failure results in incomplete vascular remodeling, contributing to placental hypoxia and oxidative stress, and ultimately contributing to the release of placental-derived factors into the maternal circulation that promote systemic endothelial dysfunction and hypertension. Also, this disease is characterized by increased metabolic dysfunctions during pregnancy [2,3]. Current evidence strongly implicates placental dysfunction as the central pathological driver of the disease, with defective trophoblast invasion, aberrant immune responses, and an imbalance between angiogenic and anti-angiogenic factors playing major roles [4,5].

The pathogenesis of PE is initiated by the inadequate remodeling of the maternal spiral arteries, leading to placental ischemia and oxidative stress. This results in the release of various vasoactive and pro-inflammatory mediators into the maternal circulation, which contribute to endothelial dysfunction, a hallmark of PE [4]. Elevated levels of soluble fms-like tyrosine kinase-1 (sFlt-1), an anti-angiogenic factor, antagonize vascular endothelial growth factor (VEGF) and placental growth factor (PlGF), impairing vascular function [6]. This angiogenic imbalance, combined with placental ischemia, creates a pro-inflammatory and oxidative environment that exacerbates maternal vascular dysfunction [7]. In parallel, the excessive production of reactive oxygen species (ROS) within the placenta exacerbates oxidative stress, leading to trophoblast apoptosis, mitochondrial dysfunction, and an inflammatory environment that further disrupts placental homeostasis [8]. Another hallmark of PE is dysregulated immune responses contributing significantly to PE pathogenesis. This immune imbalance results in the heightened activation of innate immune cells, including macrophages and neutrophils, which produce excessive levels of inflammatory cytokines such as IL-6, TNF-α, and IL-1β. These cytokines not only impair trophoblast function but also exacerbate endothelial dysfunction, creating a feed-forward loop that perpetuates disease progression [9].

Among the emerging molecular regulators of PE, growth arrest-specific protein 6 (Gas6) and its receptor AXL have gained attention due to their critical role in immune regulation, angiogenesis, and cell survival [10]. Gas6 is a vitamin K-dependent ligand that primarily signals through the TAM receptor family (Tyro3, AXL, and MerTK), with AXL being its primary receptor in endothelial and immune cells. Dysregulated Gas6/AXL signaling has been implicated in numerous inflammatory and thrombotic disorders, raising the possibility that it plays a central role in the endothelial dysfunction and immune dysregulation observed in PE [11]. Dysregulated Gas6/AXL signaling disrupts placental function by impairing trophoblast invasion, intensifying inflammation, and increasing oxidative stress—key hallmarks of preeclampsia [12]. Recent studies have demonstrated that Gas6 amplifies vascular dysfunction by promoting endothelin-1 expression, a potent vasoconstrictor, while simultaneously reducing nitric oxide (NO) bioavailability, leading to increased vascular resistance and hypertension. Furthermore, elevated plasma levels of Gas6 and soluble TAM receptor components, particularly AXL, have been observed in severe cases of PE. These levels were significantly correlated with markers of inflammation, hypertension, and proteinuria, emphasizing the role of Gas6/AXL signaling in the systemic manifestations of the disease [12,13,14,15]. Recent experimental models have demonstrated that Gas6 administration in pregnant rats induces PE-like symptoms, including hypertension and proteinuria. Conversely, the inhibition of AXL using small-molecule inhibitors, such as R428, has shown promising therapeutic potential, significantly reducing the severity of PE symptoms. These effects are mitigated by AXL inhibition using small-molecule inhibitors such as R428, suggesting that the Gas6/AXL axis is a viable target for therapeutic intervention [12]. The involvement of Gas6 in mitochondrial dysfunction is also notable, as impaired mitochondrial bioenergetics have been observed in Gas6-treated placentae, leading to an energy crisis that exacerbates trophoblast dysfunction and oxidative stress [12]. These findings underscore the significance of this pathway in the pathogenesis of PE. Our study aims to elucidate the molecular mechanisms through which Gas6 contributes to PE pathogenesis using transcriptomic, proteomic, and metabolomic approaches in a rat model. By identifying key gene targets influenced by Gas6, we hope to uncover novel therapeutic strategies that can be leveraged to improve maternal and fetal outcomes in PE. Despite these insights, the precise molecular mechanisms through which Gas6 contributes to PE remain unclear. This study aims to bridge that gap by using transcriptomic, proteomic, and metabolomic approaches in a rat model of PE.

## 2. Methods

### 2.1. Animals and Tissue Collection

This study was approved by the Brigham Young University Institutional Animal Care and Use Committee (IACUC; Approval number PRE21-0012). Pregnant Holtzman Sprague Dawley (HSD) rats, each weighing approximately 400 g, were used. On gestational day 18.5 (dGA), animals were euthanized, and both placental and fetal weights were recorded. Placental and lung tissues were harvested, with samples designated for protein analysis being snap-frozen in liquid nitrogen. Placenta tissue intended for immunofluorescence (IF) analysis was fixed via paraformaldehyde (PFA), followed by processing, embedding, and sectioning. All collected tissue samples were stored at −80 °C until further use.

### 2.2. Animal Treatment Protocols

A preeclampsia (PE) model was established using a protocol previously validated in our laboratory [12]. Pregnant rats received intraperitoneal (i.p.) injections of recombinant Gas6 protein (R&D Systems, Minneapolis, MN, USA; cat no. 8310-GS) starting on day 7.5 dGA at a dose of 4 μg/kg of body weight. Injections were administered daily for 11 days, up to day 17.5 dGA (Gas6 group, n = 10). The control group consisted of pair-fed animals receiving saline injections (n = 10).

### 2.3. Blood Pressure Measurement

Blood pressure was monitored using the CODA tail-cuff blood pressure system (Kent Scientific Corporation, Torrington, CT, USA), equipped with a heating pad and automated occlusion tail cuff. Rats were restrained for 5 min in a manufacturer-provided chamber during measurement. Blood pressure data were collected daily from all experimental groups (control and Gas6).

### 2.4. Assessment of Proteinuria

Urinary protein levels were measured using Siemens Uristix^®^ dipsticks (Siemens, Malvern, PA, USA; cat no. 1554;) according to the manufacturer’s guidelines. Urine was collected at the time of necropsy and categorized as negative, trace, +1 (30 mg/dL), +2 (100 mg/dL), +3 (300 mg/dL), or +4 (≥2000 mg/dL). Proteinuria indicative of PE was identified by readings of +3 or +4. Each experimental group included 10 animals for this analysis.

### 2.5. RNA Isolation and Extraction

Total RNA from placental tissue was extracted using the Direct-zol RNA MiniPrep kit with TriReagent (Zymo Research, Irvine, CA, USA; cat no. R2053-A). In summary, the placental tissue was homogenized at high speed in TriReagent, followed by centrifugation. The resulting supernatant was combined with an equal volume of 100% ethanol and transferred to a Zymo-Spin IICR column. DNase I treatment was conducted according to the manufacturer’s instructions, and all subsequent column washes were performed as per the kit protocol. RNA was eluted using 25 µL of DNase/RNase-Free Water and stored at −80 °C until it was ready for library preparation. mRNA was isolated from the samples using poly-dT oligo-attached magnetic beads to prepare for cDNA synthesis. The synthesis process included first-strand cDNA generation with random hexamer primers, second-strand cDNA synthesis, end repair, A-tailing, Illumina adapter ligation, size selection, amplification, and purification. Paired-end 150 base-pair reads were subsequently generated using an Illumina HiSeq 2500 instrument.

### 2.6. RNA Sequencing Analysis

RNA sequencing reads were then preprocessed and analyzed using the Galaxy analysis platform [16]. Briefly, this pipeline incorporates quality control with fastQC [Babraham bioinformatics, Babraham Institute, Cambridge, CB22 3AT], trimming with Trim Galore [Babraham bioinformatics, Babraham Institute, Cambridge, CB22 3AT], mapping and quantification with Salmon [17] using the Rattus_norvegicus.mRatBN7.2 assembly (release 112), gene-level summarization using tximport [18], and differential expression analysis with edgeR [19].

Human orthologs for the rat genes were retrieved using the homologene package in R (version 4.3.3). The statistical enrichments of Reactome intracellular signaling pathways, as well as the relevant therapeutic drugs, were retrieved using the gene symbols of the human orthologs through the enrichR platform [20,21]. Similarly, relevant drugs were also predicted using the Enrichr web-based software (https://maayanlab.cloud/Enrichr/) with the Multi-marker Analysis of GenoMic Annotation (MAGMA) and Illuminating Druggable Genome (IDG) datasets [22,23].

### 2.7. Immunofluorescence

Paraffin-embedded placental sections (n = 5) were used for immunofluorescence (IF) studies, as previously performed in our laboratory [12]. In summary, after blocking, placental slides were incubated overnight with a rabbit primary antibody against FAM11a or Clca4. (Abcam; cat no. ab200718 or cat no. ab197347). The next day, a secondary donkey anti-rat Texas Red (TR; Santa Cruz Biotechnology, Santa Cruz, CA, USA) was incubated for an hour. IF detection was performed using a BX6 microscope.

### 2.8. Statistical Analysis

Significant changes in proteinuria and blood pressure were determined using an unpaired Student’s T-test (*p*-value < 0.05). Significance in differentially expressed genes in the RNA-sequencing data was determined with a negative binomial test implemented in edgeR [19] after a false discovery rate (FDR) correction to adjust for multiple-hypothesis testing (FDR-adjusted *p*-value < 0.05). The significances for the intracellular signaling pathways and drugs were calculated using default enrichR parameters.

## 3. Results

### 3.1. Gas6 Induces Preeclampsia (PE) in Rats

**Figure 1:**
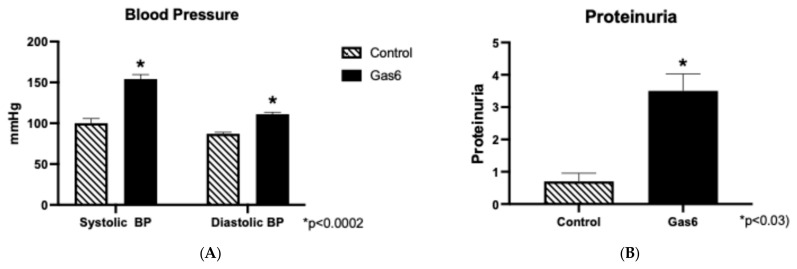
The blood pressure and proteinuria of Gas6-treated animals. There was a significant increase in both systolic diastolic pressure (**A**) and urine proteinuria (+3 and +4) (**B**) in Gas6-treated animals as compared to controls (n = 10).

Preeclampsia (PE) was induced in pregnant rats through an 11-day treatment with Gas6. The treatment led to significant increases in both systolic and diastolic blood pressure in the rats, as measured at necropsy (Figure 1A). No differences were observed in placenta and fetal weights. Proteinuria levels were also evaluated, revealing a marked increase (+3 to +4) in Gas6-treated rats compared to controls (Figure 1B).

### 3.2. Differential Expression of Rat Genes

To determine Gas6-induced transcriptional changes, we analyzed intracellular gene expression in Gas6-treated and control animals. To do so, we generated bulk RNA-sequencing data from both sets of animals. We then pre-processed these RNA-sequencing reads using well established tools for quality control, trimming, mapping and quantification, as well as differential expression.

**Figure 2:**
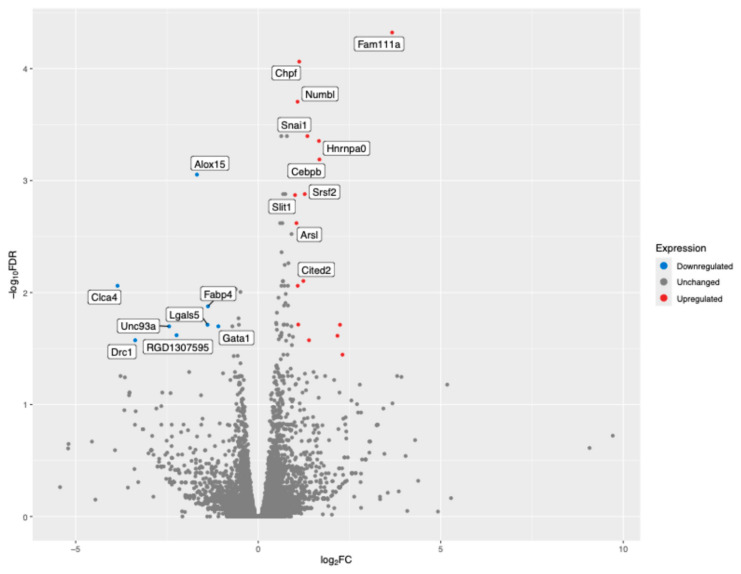
A Volcano plot of the most significant differentially expressed genes in the Gas6 vs. healthy control comparison. The top 18 genes (10 up-regulated and 8 down-regulated) that met the thresholds of FDR-adjusted *p*-value < 0.05 and log_2_ fold-change >1.0 are labeled. Red and blue dots represent up-regulated and down-regulated genes, respectively.

We detected transcripts from more than 21,000 genes, of which 98 were significantly differentially expressed with a false-discovery rate *p*-value less than 0.05. Only 25 genes remained after imposing an additional threshold of an absolute log2 fold-change value greater or equal to one (Figure 2).

The significant up-regulated rat genes with the most positive log_2_ fold-change values included FAM111 trypsin-like peptidase A (Fam111a), heat shock protein family A member 1-like (Hspa1l), and cyclin-dependent kinase inhibitor 2A (Cdkn2a) (Table 1). In contrast, the significant down-regulated genes with the most negative log2 fold-change values included chloride channel accessory 4 (Clca4) and dynein regulatory complex subunit 1 (Drc1).

### 3.3. Significant Signaling Pathways

Given the lack of characterized signaling pathways in rat cells, we retrieved the human orthologs for the differentially expressed rat genes with an FDR-adjusted *p*-value < 0.05. We obtained more rat genes with human homologs than what we had expected (82 of 98), which was still sufficient to enable pathway enrichment analysis.

The enrichment results can be classified into three broader categories, including cellular structure (e.g., extracellular matrix, collagen, laminin, hemidesmosome, and cell junctions), metabolism (e.g., eicosatetraenoic acid, chondroitin sulfate, and carbohydrates), and intercellular signaling (e.g., interleukin signaling, and cell–cell communication) (Table 2).

### 3.4. Immunofluorescence Validation of FAM111A and Clca4

**Figure 3:**
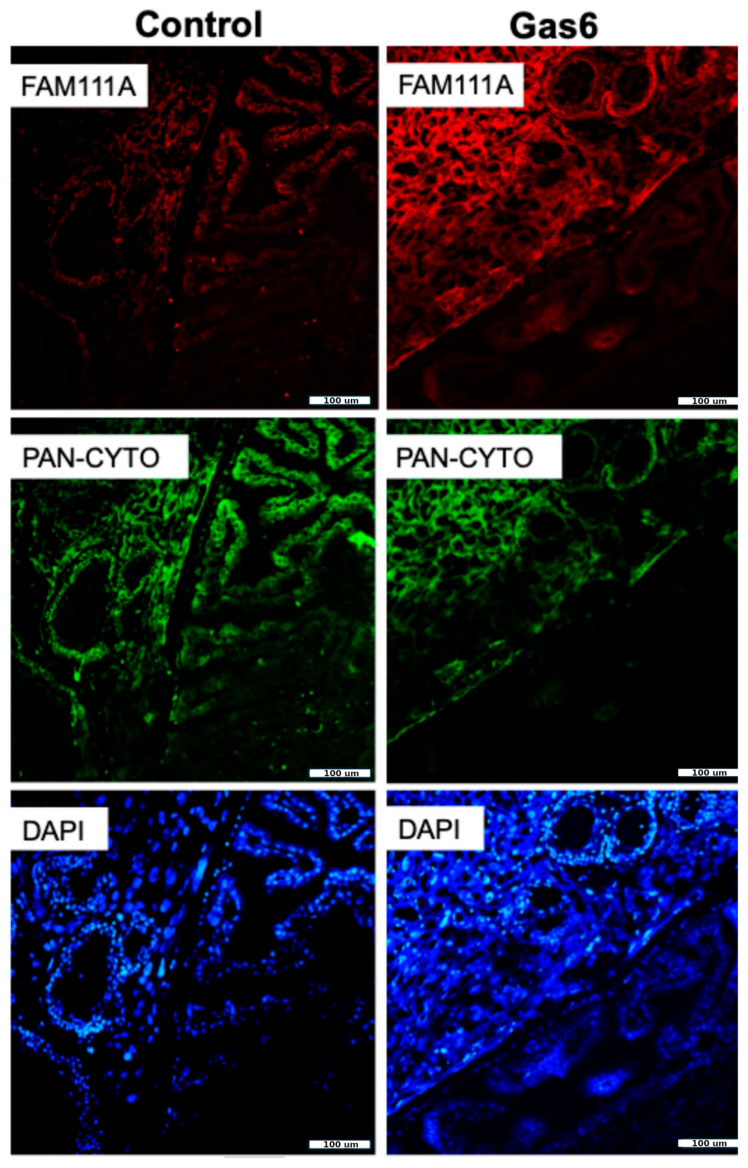
Placental FAM111A protein in Gas6 and control animals. Placental FAM111A staining was performed between control and Gas6 placentae. Increased FAM111A protein levels were observed in placentae of Gas6 animals as compared to controls.

**Figure 4:**
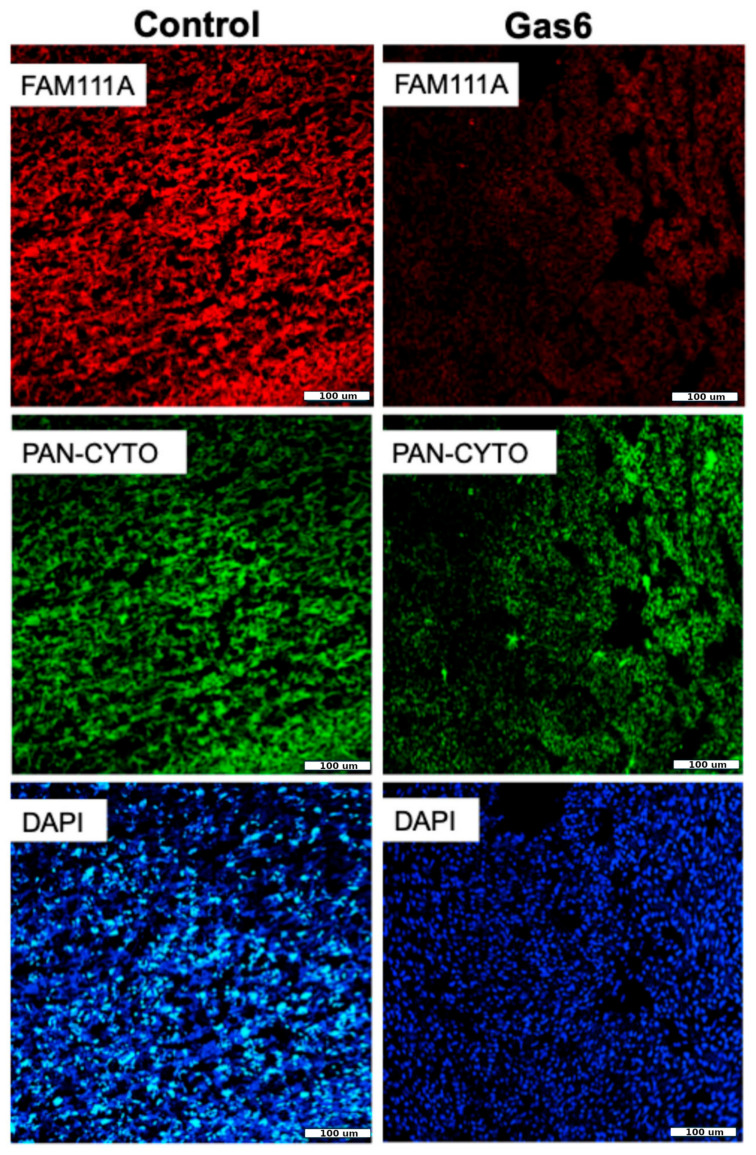
Placental Clca4 protein in Gas6 and control animals. Clca4 staining was performed between control and Gas6 placentae. Clca4 protein levels were decreased in placentae of Gas6 animals as compared to controls.

To validate the RNA seq results observed, we chose to histologically determine the protein expression of the highest increased gene (FAM111A) and highest decreased gene (Clca4) between the control and Gas6 placentae. Histological assessment of the placentae revealed that FAM111A protein expression was increased in the animals treated with Gas6 as compared with the controls. (Figure 3). In contrast, Clca4 protein was decreased in the animals treated with Gas6 as compared with the controls (Figure 4).

### 3.5. Gene Human Orthologs an Associated Drugs Agent

We next attempted to determine whether any existing small-molecule drugs could potentially be repurposed as therapeutics against preeclampsia. To do so, we enriched the human homologs of the differentially expressed genes, from the comparison between Gas6 vs. healthy control animals, against datasets containing gene/drug associations. The results of this analysis provided a list of 31 small molecules that could be relevant for preeclampsia in the clinic (Table 3). We noticed that some of these potential therapeutics are known anti-inflammatory agents, while others likely have a more targeted mechanism of action that could contribute to PE treatment.

## 4. Discussion

Preeclampsia (PE) remains a complex pregnancy-specific disorder characterized by hypertension, proteinuria, and systemic inflammation. The involvement of Gas6 in PE has gained attention due to its ability to activate the AXL receptor, which is implicated in cellular processes such as survival, proliferation, migration, and inflammation. Elevated plasma levels of Gas6 have been correlated with severe PE cases, reinforcing its systemic impact on maternal and placental pathophysiology [15,24]. Experimental evidence supports that Gas6 administration induces hallmark PE symptoms, including elevated blood pressure and proteinuria, with AXL inhibitors like R428 showing potential in mitigating these effects [12].

This study provides compelling evidence that Gas6 is a pivotal mediator of PE pathophysiology, acting through multiple mechanisms to induce placental oxidative stress, inflammation, and extracellular matrix remodeling. The transcriptomic analysis performed in this study revealed significant changes in gene expression that contribute to PE progression, many of which are directly modulated by Gas6 activity. The upregulation of Fam111a suggests its involvement in oxidative stress and trophoblast dysfunction. Fam111a, linked to cellular stress responses and DNA replication, may exacerbate oxidative damage in the placenta, a key feature of PE [25,26]. Conversely, the downregulation of Clca4, a gene involved in ion transport, highlights potential disruptions in cellular signaling pathways critical for placental homeostasis and nutrient exchange [25,26]. Together, these findings align with earlier studies that emphasize the role of oxidative stress and inflammation in PE-related placental dysfunction [9,27,28].

The enrichment analysis of differentially expressed genes identified pathways related to extracellular matrix (ECM) organization, interleukin signaling, and oxidative stress, which are central to PE pathophysiology. Aberrant ECM remodeling, as observed in Gas6-treated placentae, impairs trophoblast invasion and spiral artery remodeling, leading to placental ischemia and hypoxia [29,30,31]. Additionally, interleukin signaling pathways involving cytokines like IL-6 and IL-8 contribute to systemic inflammation and endothelial dysfunction, worsening PE outcomes [32,33,34]. The observed oxidative stress pathways further validate the role of Gas6 in amplifying placental dysfunction through increased reactive oxygen species (ROS) production. These findings reinforce that Gas6-driven changes in placental structure and function are key contributors to the onset and progression of PE. The identification of Gas6 as a central player in PE pathogenesis has significant therapeutic implications. Our study highlights the potential of AXL-specific inhibitors and Gas6-neutralizing antibodies as promising therapeutic strategies to mitigate PE symptoms. Preclinical models demonstrate that AXL inhibitors, including small molecules and monoclonal antibodies, effectively reduce hypertension, proteinuria, and placental inflammation [12]. These findings are consistent with clinical observations, where interventions targeting angiogenic imbalance and oxidative stress alleviate PE symptoms [32,33]. Combining AXL inhibition with existing therapies aimed at restoring angiogenic balance could offer a multifaceted approach to managing PE. Future investigations should aim to refine these therapeutic strategies, assessing their safety and efficacy in human pregnancies.

The immunofluorescence validation of FAM111A and Clca4 further underscores the relevance of transcriptomic findings. Elevated FAM111A levels in Gas6-treated placentae highlight its potential as a biomarker for oxidative stress and cellular dysfunction. Meanwhile, reduced Clca4 expression underscores disruptions in chloride ion transport and cellular homeostasis, crucial for maintaining placental integrity [26,35].

Interestingly, at least one subset of the existing small molecules that were predicted to be relevant for PE have been evaluated in prior studies. Specifically, the aldehyde dehydrogenase mechanism of action for disulfiram has promise in reducing PE, though it has achieved mixed results in the clinic [36]. Quercetin, which is an antioxidant, has been shown to decrease multiple clinical symptoms of PE [37]. Similarly, treatment with chlorpromazine substantially blocked STBEV uptake and prevented placental alkaline phosphatase (PLAP)-positive staining on the plasma membrane of endothelial cells, which partially reduced PE symptoms [37,38]. These prior findings at least partially support the human relevance of our Gas6 rat model of PE, but still necessitate additional follow-up validation experiments.

The findings of this study provide a strong foundation for understanding the molecular mechanisms underpinning Gas6-mediated placental dysfunction in PE. Additionally, the identification of gene targets associated with Gas6 dysregulation provides an opportunity for biomarker development. While our study provides novel insights, further research is needed to explore the broader implications of Gas6 signaling in human PE.

Longitudinal studies evaluating the correlation between Gas6 plasma levels and PE severity across different populations could enhance our understanding of its predictive value in clinical settings. Moreover, combining AXL inhibitors with existing treatments, such as aspirin or antioxidants, may offer a synergistic approach to mitigating PE symptoms.

In conclusion, this study highlights Gas6 as a pivotal regulator of PE pathophysiology, with transcriptomic evidence supporting its involvement in oxidative stress, inflammation, and trophoblast dysfunction. Our findings reinforce the therapeutic potential of Gas6/AXL inhibition, while also highlighting new molecular targets associated with placental metabolic dysregulation. The therapeutic potential of targeting the Gas6/AXL axis represents a promising avenue for the development of novel treatments for PE, ultimately improving maternal and fetal health outcomes. Given the global burden of PE and its long-term consequences on maternal and fetal health, a deeper understanding of Gas6-mediated mechanisms may provide transformative insights for developing precision medicine approaches in obstetric care.

## Figures and Tables

**Table 1:** Top 25 differentially expressed genes from Gas6 vs. healthy control comparison, ranked by statistical significance.

Gene Symbol	Gene Description	Log_2_FC	FDR
Fam111a	FAM111 trypsin-like peptidase A	3.67	4.76 × 10^−5^
Hspa1l	heat shock protein family A (Hsp70) member 1-like	2.31	3.59 × 10^−2^
Cdkn2a	cyclin-dependent kinase inhibitor 2A	2.31	8.70 × 10^−3^
Bloc1s3	biogenesis of lysosomal organelles complex-1, subunit 3	2.24	1.94 × 10^−2^
H2ax	H2A.X variant histone	2.17	2.43 × 10^−2^
Cebpb	CCAAT/enhancer binding protein beta	1.68	6.48 × 10^−4^
Hnrnpa0	heterogeneous nuclear ribonucleoprotein A0	1.66	4.43 × 10^−4^
Comtd1	Catechol-O-methyltransferase domain containing 1	1.39	2.67 × 10^−2^
Snai1	snail family transcriptional repressor 1	1.35	4.01 × 10^−4^
Srsf2	serine and arginine rich splicing factor 2	1.27	1.32 × 10^−3^
Cited2	Cbp/p300-interacting transactivator, with Glu/Asp-rich carboxy-terminal domain, 2	1.24	7.88 × 10^−3^
Chpf	chondroitin polymerizing factor	1.12	8.66 × 10^−5^
Mrps12	mitochondrial ribosomal protein S12	1.10	1.93 × 10^−2^
Phlda1	pleckstrin homology-like domain, family A, member 1	1.08	8.70 × 10^−3^
Numbl	NUMB-like endocytic adaptor protein	1.08	1.97 × 10^−4^
Arsl	arylsulfatase L	1.05	2.40 × 10^−3^
Slit1	slit guidance ligand 1	1.01	1.35 × 10^−3^
Gata1	GATA binding protein 1	−1.09	2.00 × 10^−2^
Fabp4	fatty acid binding protein 4	−1.38	1.33 × 10^−2^
Lgals5	galectin 5	−1.39	1.94 × 10^−2^
Alox15	arachidonate 15-lipoxygenase	−1.68	8.84 × 10^−4^
RGD1307595	similar to RIKEN cDNA 1700018B24	−2.24	2.40 × 10^−2^
Unc93a	Unc-93 homolog A	−2.44	2.00 × 10^−2^
Drc1	dynein regulatory complex subunit 1	−3.37	2.67 × 10^−2^
Clca4	chloride channel accessory 4	−3.85	8.70 × 10^−3^

**Table 2:** Significant intracellular signaling pathways from Gas6 vs. healthy control comparison, ranked by statistical significance using Reactome.

Term	Adjusted *p*-Value	Genes
Extracellular Matrix Organization	8.04 × 10^−4^	*LAMA5*; *COL4A2*; *ITGB4*; *ACTN1*; *SERPINH1*; *AGRN*; *CAPN15*; *LOXL2*; *PLEC*
Non-Integrin Membrane-ECM Interactions	8.04 × 10^−4^	*LAMA5*; *COL4A2*; *ITGB4*; *ACTN1*; *AGRN*
Collagen Formation	4.26 × 10^−3^	*COL4A2*; *ITGB4*; *SERPINH1*; *LOXL2*; *PLEC*
Assembly of Collagen Fibrils and Other Multimeric Structures	9.68 × 10^−3^	*COL4A2*; *ITGB4*; *LOXL2*; *PLEC*
Cell Junction Organization	9.68 × 10^−3^	*JUP*; *ITGB4*; *ACTN1*; *SNAI1*; *PLEC*
Synthesis of 15-Eicosatetraenoic Acid Derivatives	1.33 × 10^−2^	*GPX2*; *ALOX15*
Laminin Interactions	1.33 × 10^−2^	*LAMA5*; *COL4A2*; *ITGB4*
Synthesis of 12-Eicosatetraenoic Acid Derivatives	1.60 × 10^−2^	*GPX2*; *ALOX15*
Cell–Cell Communication	1.94 × 10^−2^	*JUP*; *ITGB4*; *ACTN1*; *SNAI1*; *PLEC*
Type I Hemidesmosome Assembly	3.33 × 10^−2^	*ITGB4*; *PLEC*
Chondroitin Sulfate Dermatan Sulfate Metabolism	3.52 × 10^−2^	*CHPF*; *CHPF2*; *AGRN*
Interleukin-4 and Interleukin-13 Signaling	3.52 × 10^−2^	*LAMA5*; *NOS2*; *ALOX15*; *FSCN1*
Metabolism of Carbohydrates	3.52 × 10^−2^	*GYS1*; *CHPF*; *CHPF2*; *MAN2B2*; *AGRN*; *GALK1*

**Table 3:** Significant drug/gene interactions in human orthologs from Gas6 vs. healthy control comparison.

Agent	*p*-Value	Odds Ratio	Combined Score	Genes	Source Database
Disulfiram	0.0096	14.62	67.99	*ALOX15*; *LOXL2*	IDG
Quercetin	0.0129	6.48	28.19	*NOS2*; *ALOX15*; *CALM2*	IDG
Stearic Acid	0.0203	61.46	239.43	*FABP4*	IDG
Monobenzone	0.0203	61.46	239.43	*ALOX15*	IDG
Methyldopa	0.0244	49.17	182.67	*ALOX15*	IDG
Hydrocortisone	0.0244	49.17	182.67	*NOS2*	IDG
Chlorzoxazone	0.0244	49.17	182.67	*NOS2*	IDG
Levodopa	0.0284	40.97	145.98	*ALOX15*	IDG
Zileuton	0.0323	35.12	120.50	*ALOX15*	IDG
Estriol	0.0323	35.12	120.50	*ALOX15*	IDG
Estriol Succinate	0.0323	35.12	120.50	*ALOX15*	IDG
Aprindine	0.0363	30.73	101.88	*CALM2*	IDG
Thiram	0.0363	30.73	101.88	*LOXL2*	IDG
Mitotane	0.0403	27.31	87.73	*FDX1*	IDG
Rifampicin	0.0403	27.31	87.73	*ALOX15*	IDG
Thioguanine	0.0403	27.31	87.73	*ALOX15*	IDG
Etoposide	0.0403	27.31	87.73	*ALOX15*	IDG
Chlorpromazine	0.0427	6.36	20.05	*ALOX15*; *CALM2*	IDG
Hydralazine	0.0442	24.58	76.66	*ALOX15*	IDG
Estrone	0.0481	22.34	67.79	*ALOX15*	IDG
Sirolimus	0.0481	22.34	67.79	*FKBP3*	IDG
Hydrochlorothiazide	0.0123	12.74	56.07	*DOT1L*; *SLIT1*	MAGMA
Thiazide Diuretic	0.0123	12.74	56.07	*DOT1L*; *SLIT1*	MAGMA
Hexachlorophene	0.0134	12.12	52.22	*ALOX15*; *GALK1*	MAGMA
Triflusal	0.0203	61.46	239.43	*NOS2*	MAGMA
Ethanolamine Oleate	0.0203	61.46	239.43	*FABP4*	MAGMA
Tacrine	0.0244	49.17	182.67	*ALOX15*	MAGMA
Nonivamide	0.0244	49.17	182.67	*ALOX15*	MAGMA
Dienestrol	0.0284	40.97	145.98	*ALOX15*	MAGMA
Candesartan	0.0363	30.73	101.88	*DOT1L*	MAGMA
L Arginine	0.0403	27.31	87.73	*NOS2*	MAGMA

## Data Availability

The original contributions presented in this study are included in the article. Further inquiries can be directed to the corresponding author.
